# A Manual of Surgical Treatment

**Published:** 1904-03

**Authors:** 


**A Manual of Surgical Treatment.** By W. Watson Cheyne, C.B., M.B., and F. F. Burghard, M.D. Part VI., Section II. Pp. xxiii., 559. London: Longmans, Green, and Co. 1903.

This volume, which is one of the largest of the series, is also perhaps the best. The subjects treated of are of great importance, and are very ably dealt with. Our information with regard to many has greatly advanced during recent years, and such advance is fully brought under our notice in this work. This volume contains an account of many affections within the abdomen, and of genito-urinary surgery and other important subjects. The authors acknowledge their indebtedness to the recent work of Henry Morris in connection with the surgery of the kidney and ureter, and to Fowler and Godlee's work on diseases of the chest. Much valuable information has also been gathered from Quénu and Hartmann's work on the surgery of the rectum. The illustrations are as remarkably good as they were in the chapters on abdominal surgery in the last volume, particularly those illustrating diseases of the rectum and the kidney.

We think, in connection with the subject of enlargement of the spleen and its treatment by splenectomy, the important condition of splenic anæmia and its operative treatment might have found a place.

It is interesting to note the opinion of the authors on the value of oöphorectomy in cancer of the breast. They have evidently a poor estimate of it, for they only advise its performance if the disease can at the same time be almost completely removed; and even in such condition they admit they have seen striking instances of the futility of the proceeding, and they say that they have never known an actual cure.

As this is the last volume of the series, we must say how greatly we value them as a whole, and how strongly we advise their addition to the library of every practitioner.

